# The effect of quercetin and citrulline on cycling time trial performance

**DOI:** 10.1080/15502783.2024.2416909

**Published:** 2024-10-17

**Authors:** Jennifer A. Kurtz, Jacob Grazer, Kathryn Wilson, Rafaela G. Feresin, J. Andrew Doyle, Ryan Middleton, Emma Devis, Trisha A. VanDusseldorp, Kimberly Fasczewski, Jeff Otis

**Affiliations:** a Appalachian State University, Department of Public Health & Exercise Science, Boone, NC, USA; b Georgia State University, Department of Kinesiology & Health, Atlanta, GA, USA; c Kennesaw State University, Department of Exercise Science and Sport Management, Kennesaw, GA, USA; d Georgia State University, Center for the Study of Stress, Trauma, and Resilience, Atlanta, Georgia; e Georgia State University, Department of Nutrition, Atlanta, GA, USA; f University of Miami, Miller School of Medicine, Department of Physical Therapy, Coral Gables, FL, USA; g LLC p/b JDS Therapeutics, Bonafide Health, Harrison, NY, USA

## Abstract

**Background:**

There is growing interest in the use of nutrition and dietary supplements to optimize training and time-trial (TT) performance in cyclists. Separately, quercetin (QCT) and citrulline (CIT) have been used as ergogenic aids to improve oxygen (VO_2_) kinetics, perceived effort, and cycling TT performance. However, whether the combination of QCT and CIT can provide additive benefits and further enhance cycling performance production is currently unknown.

**Methods:**

We examined 28-days of QCT + CIT supplementation on TT performance and several performance measures (i.e. mean power, VO_2_, respiratory exchange ratio (RER), and rate of perceived exertion (RPE)). Forty-eight highly trained cyclists were assigned to one of four supplementation groups: (1) QCT + CIT (QCT: 500 mg, CIT: 3000 g), (2) QCT (500 mg), (3) CIT (3000 mg), or (4) placebo (3500 mg of a zero-calorie flavored crystal light package). Supplements were consumed two times per day for 28 consecutive days. Participants performed a 20-km cycling time-trial race, pre- and post-supplementation to determine the impact of the combined effects of QCT + CIT.

**Results:**

There were no potential benefits of QCT +CIT supplementation on TT performance and several performance measures. However, there was an improvement in VO_2_ from pre-to-post-supplementation in QCT (*p*  = 0.05) and CIT (*p*  = 0.04) groups, but not in the QCT+CIT and PL groups.

**Conclusions:**

QCT + CIT does not seem beneficial for 20-km TT performance; further exploration with a focus on an increase in cycling duration or QCT+CIT combined with additional polyphenols may amplify any perceived bioactive or metabolic effects on cycling performance. The efficacy of QCT + CIT supplementation to improve cycling performance remains ambiguous.

## 1. Background

There is growing interest in cycling to optimize training and time-trial (TT) performance using pharmaceutical or nutritional supplementation strategies. Due to the competitive nature of TTs races, ergogenic aids have become more popular. Nutritional ergogenic supplements may increase oxygen consumption and improve ATP production [1,2] decrease fatigue, improve cycling efficiency and power output, or assist in recovery during intense training [3]. While few studies have looked at the ergogenic effects of quercetin (QCT) and citrulline (CIT) separately on cycling performance, to the best of our knowledge; no studies have examined the ergogenic effects of co-supplementation. As the popularity and competitiveness of TTs increase, we seek to provide evidence that the co-supplementation of QCT + CIT will provide a safe and effective nutritional strategy to improve cycling VO_2_ kinetics and performance.

QCT is a plant pigment, an antioxidant, a polyphenol, and a flavanol belonging to the flavonoid group [4,5]. QCT is a powerful antioxidant and anti-inflammatory compound commonly found in apples, elderberries, citrus fruits, red wine, red onions, hot peppers, berries, kale, buckwheat tea, dark green leafy greens, and capers [5–7]. Recently, QCT has received attention due to its effects on improving oxygen consumption and VO_2_ kinetics [8,9]. However, there is modest evidence to suggest QCT improves cycling performance in trained individuals [10–12]. These multifaceted metabolic effects of QCT combined with other molecules make it a potential ergogenic aid to improve endurance performance. However, the effect of the combination of a polyphenol (QCT) with an amino acid is unknown on endurance performance.

Citrulline (CIT) is a nonessential amino acid found in high concentrations in watermelon [13–15]. CIT is formed from arginine, an amino acid involved in several physiological roles including the urea cycle, protein synthesis, and from the activity of nitric oxide synthase enzymes yielding nitric oxide [15–20]. CIT can be formed through the activity of nitric oxide synthase enzymes yielding NO [16]. Emerging evidence suggests that CIT can be used as an ergogenic aid since it has strong antioxidant [16,21] and anti-inflammatory properties [17,22], promotes muscle protein synthesis [23,24], skeletal muscle metabolism, and mitochondrial biogenesis [19], oxygen uptake [25–27], post-exercise muscle function, RPE, and recovery effects [28].

Several lines of research suggest when provided separately, that both QCT and CIT may improve components of cardiovascular health, metabolism, oxidative stress, and performance as an ergogenic aid by possible synergistic interactions. For example, a dose of 6 or 8 g of L-citrulline modestly improved endurance cycling performance in trained individuals [29,30]. Additionally, 1000 mg/day of QCT combined with other ingredients positively improved performance [9–12,31–33]. However, to date, no studies exist investigating QCT + CIT’s potential synergistic roles on energy metabolism, endurance performance capacity, VO_2_ kinetics, and cycling performance. It is unknown what physiological mechanisms QCT and CIT target to improve cycling performance. To address these gaps in knowledge, the present study tested the ergogenic effects of daily consumption of QCT + CIT, QCT, CIT, or placebo (PL) for 28 consecutive days on cycling TT performance variables (i.e. average power, VO_2_, respiratory exchange ratio (RER), and rate of perceived exertion (RPE)). We hypothesized that QCT + C will provide additional performance advantages compared to QCT and CIT alone. We aimed to assess QCT + CIT as a novel, safe, and effective nutritional strategy and to investigate its effects on cycling cardiovascular changes and aerobic performance in trained cyclists.

## 2. Methods

### 2.1. Participants

Participants included male and female cyclists who regularly competed in races (mountain, gravel, cross country, road, cyclocross). Cyclists were defined as Tier 2 of a 6-Tier framework to classify exercise/training and/or sports performance levels [34]. Tier 2 is defined as a trained, developed individual who identified cycling as their main sport [35] and provided a sport-specific metric of training volume (average 101.58 ± 64.36 - 285.92 ± 92.10 km/week and 11.16 ± 5.08 - 18.55 ± 7.41 hrs/week). The participants were recruited through local cycling teams and races and trained at least three times per week, currently trained with a stationary bike/trainer, trained at least 3 to 5 hours per week over the past 3 years [36,37] and trained with a purpose to compete [35,36,38]. All females were tested during their follicular phase (approximately day 0 to day 16, assuming a 30-day regular cycle [39]), where female sex hormone concentrations are relatively stable and most similar to other women [40]. We included females with medically prescribed monophasic or biphasic oral contraceptives, perimenopausal [41], and excluded triphasic oral contraceptives due to a possible decrease in peak oxygen uptake (volume of O_2peak_ per minute) [42]. We excluded the following participants: performed greater than 2 days of resistance training per week; daily use of nonsteroidal anti-inflammatory drugs, and/or used of anti-hypertensive medications; smoker (in the past 6 months); smoked or used THC or CBD products; diagnosed with chronic, systemic, or inflammatory diseases; pregnant women; females who have not had a period in the past 6 months (i.e. amenorrhea); documented intolerance to iron; have an orthopedic injury that may impact cycling performance testing. This study was approved by and carried out in accordance with the University’s Institutional Review Board for the protection of human subjects (IRB # H23189; Approval Date: 11/04/2022)

### 2.2. Experimental design

**Figure 1.**
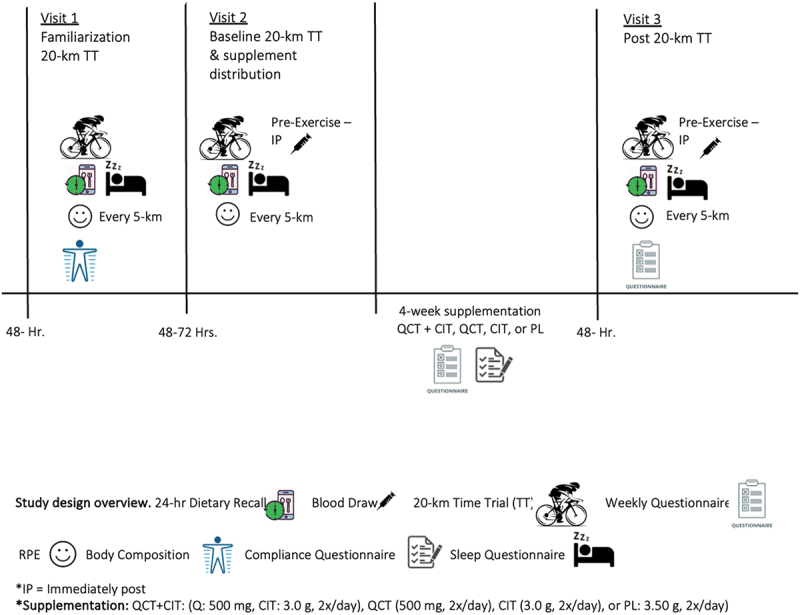
Study design overview.

We employed a randomized, double-blinded, placebo-controlled study design (Figure 1). To ensure a double-blinded study, another researcher rolled a four-sided die for randomization and managed the supplement distribution. Participants visited the Applied Exercise Physiology laboratory on three separate occasions scheduled throughout the day (0700–1600) at the same time of day (±2 hours), spanning a five-to-six-week period. Visits required participants to perform a 20-km cycling TT on three separate visits while examining average power, VO_2_, RER, and RPE. During the testing period, participants maintained their typical race training regimen but avoided strenuous exercise for at least 48 hours prior and only low-intensity exercise 24 hours prior to each visit and agreed to avoid the use of large-dose vitamin or mineral supplements (>100% of Recommended Dietary Allowances (RDA)), nutritional supplements or ergogenic aids such as QCT, CIT, creatine, B-alanine, antioxidant medications, tocopherols, or flavonoid supplementation, herbs, and anti-inflammatory or hypertensive medications during the testing periods. Participants were asked to follow a diet moderate in carbohydrates and protein similar to what they would normally consume before a race before each visit. Participants completed a 24-hour dietary recall before each visit to ensure diet replication for subsequent visits. Body weight was recorded before each visit. Visit 1 consisted of completing the informed consent, health history/medical history questionnaire, 24-hr dietary recall, injury history questionnaire, and PARQCT+, Dual Energy X-ray Absorptiometry (DEXA) body composition scan, measurement of height and weight, and a 20-km TT familiarization bout. Participants had their body composition (Lunar Prodigy encore: PR 510,021), height, and weight measured before the familiarization session. Visit 2 consisted of a baseline-TT performance bout prior to a four-week supplementation period. Visit 1 and visit 2 took place 72 hours apart, avoiding strenuous exercise for at least 48 hours. After visit 2, participants were randomly assigned a supplement. Following the 28-day supplementation period, participants returned for visit 3 for the post-20-km performance test. Once the participants finished the 20-km, they performed a self-selected 5-minute cool-down.

#### 2.2.1. 20-km time-trial performance

Participants completed a 10-minute warm-up at a self-selected pace and intensity [43,44] before the 20-km TT. The TTs were performed on a Wahoo Core Kickr Smart Trainer using the Zwift system virtual training app. The race consisted of a reproducible 20-km TT composed of flat terrain at a freely selected pedaling cadence allowing for the collection of average power, as previously described [37,38,45–47]. The KICKR Trainer (Wahoo Fitness, Atlanta, Georgia) was set in open test mode during the TT, allowing participants to change gears and intensity freely. The participants were asked to produce their maximal power output for the TT and adopt their personal pacing strategies [48–50] and were instructed to complete the total distance in the fastest time possible [45]. Participants were allowed to drink water *ad libitum* and were allowed to listen to the same playlist of music at each visit.

### 2.3. 20-km performance measurements

We collected the following performance variables: time-to-completion, average power, oxygen consumption (VO_2_), RER, heart rate (HR), and RPE (6–20 scale). All individuals wore a metabolic mask (Parvo Medics, TrueOne 2400, Salt Lake City, UT) for 2 to 3 minutes to capture oxygen consumption at each 5-km mark. We collected VO_2_ every 5-km (5, 10, 15, and 20 km) by indirect calorimetry (Parvo Medics, True One 2400 Metabolic unit, Salt Lake, City, UT) to detect their average oxygen consumption (VO_2_) and RER at each 5-km mark [51]. The highest achieved RER indicated high-intensity effort during the whole 20-km at each 5-km mark. An RER in the range between ≥1.1 and ≥1.15 and RPE ≥17 or ≥18 indicated participants neared maximal effort [52–54]. During all testing protocols, HR (bpm) was monitored using a chest heart rate monitor (Polar heart rate monitors, model H10). HR was recorded every 5 km. All individuals reported their RPE using the 6–20-point Borg scale [52,53] at each 5-km mark. The highest achieved RPE indicated high-intensity effort during the whole 20-km at each 5-km mark. Following the 20 km, a 5-minute cool-down at a self-selected intensity was performed.

#### 2.3.1. Supplementation

The participants were randomized to one of four groups: (1) QCT+CIT, (2) QCT, (3) CIT, or (4) PL. Supplements were dissolved in 16 oz of water. The composition of these powders were as follows: 1) QCT+CIT (500 mg of quercetin dihydrate, 3000 mg of L-citrulline, 3500 mg orange crystal light), 2×/day; 2) QCT (500 mg of quercetin dihydrate, 3500 mg orange crystal light), 2×/day; 3) CIT (3000 mg of L-citrulline and 3500 mg orange crystal light), 2×/day and; 4) PL (3500 mg orange crystal light), 2×/day, following visit 2. Two separate dosages of QCT and CIT were chosen to increase absorption and based on previous research in which supplements were observed to positively improve performance [8–11,25,29,31–33]. Since aerobic adaptations can be detected after 4 weeks of training in trained individuals, the four-week supplementation period was chosen [55–57]. The placebo contained a zero-calorie orange-flavored crystal light package powder dissolved in 16 oz of water, like the treatment in the supplement.

#### 2.3.2. Supplementation conditions strategy

Participants consumed their supplement twice per day after meals for 4 weeks [10,11,33] (Figure 1). Participants were instructed to add the powdered supplement to 16 oz of water and consume within 30 minutes of their first and last meals of each day. The zero-calorie orange-flavored crystal light package was added to mask any taste and ensure that participants remained blinded to their group. The supplements were dissolved in a beverage form to enhance absorption [58,59]. Participants were required to add only filtered or bottled room-temperature water to the bottle, but no other fluids were allowed in the mix. During the supplementation period, participants received a weekly phone call or text check-ins with a research team member and logged physical activity, gastrointestinal distress (GI), and supplement compliance throughout the supplementation period. To ensure consistency, participants were required to track when they consumed the supplement on a daily supplement compliance dosing diary. If participants were not compliant and missed more than 10% (~5.6 supplement bags), a sensitivity analysis was performed to see what extent it may or may not have influenced the primary outcomes of interest. Participants were required to track their physical activity on a compliance dosing diary, including their intensity (i.e. 6–20 RPE scale), mode, and duration.

#### 2.3.3. Statistical analyses

All data were reported as mean ± standard deviation or frequency (%). SPSS statistical software (V. 24.0, Chicago, IL, USA) was used for all analyses. A criterion alpha level of 0.05 was used to determine statistical significance. All data were tested for normality using the Shapiro-Wilk test. If normality assumptions were violated, an equivalent non-parametric test was performed. Descriptive statistics were reported for all study variables. Test re-test reliability was conducted on TT performance. The reproducibility was expressed using the coefficient of variation (CV [%]), and intraclass correlation coefficient (ICC) using a one-way random effects model, and change in the mean between test and re-test was calculated from visit 1, visit 2, and visit 3. We performed a 2 (pre-post supplement) × 4 (condition) mixed model ANOVA to assess mean differences in TT time to completion. A 2 (pre/post) × 4 (condition) × 5 (repeated measures [every 5-km] within bouts) mixed model ANOVA was used to assess RPE, average power, heart rate, and VO_2_ (average oxygen utilization). Additionally, we repeated all mixed model analyses excluding women as sensitivity analyses to see to what extent the inclusion of women influenced the observed effects. If statistical significance was found, data were reported. Effect sizes were expressed as partial eta squared (η^2^). Effect size thresholds were categorized and interpreted as small (η^2^ = 0.01), medium (η^2^ = 0.06), and large (η^2^ = 0.14) [60–62]. In the event of a significant F-ratio, the model was decomposed using a series of between-groups and repeated-measures ANOVAs with Bonferroni correction. In the case of unexpected protocol abnormalities (e.g. a female participant’s menstrual cycle appears abnormal) or an extension of the duration between visits is unavoidable, we performed a sensitivity analysis to determine to what extent it may have affected or changed our observations.

## 3. Results

**Figure 2.**
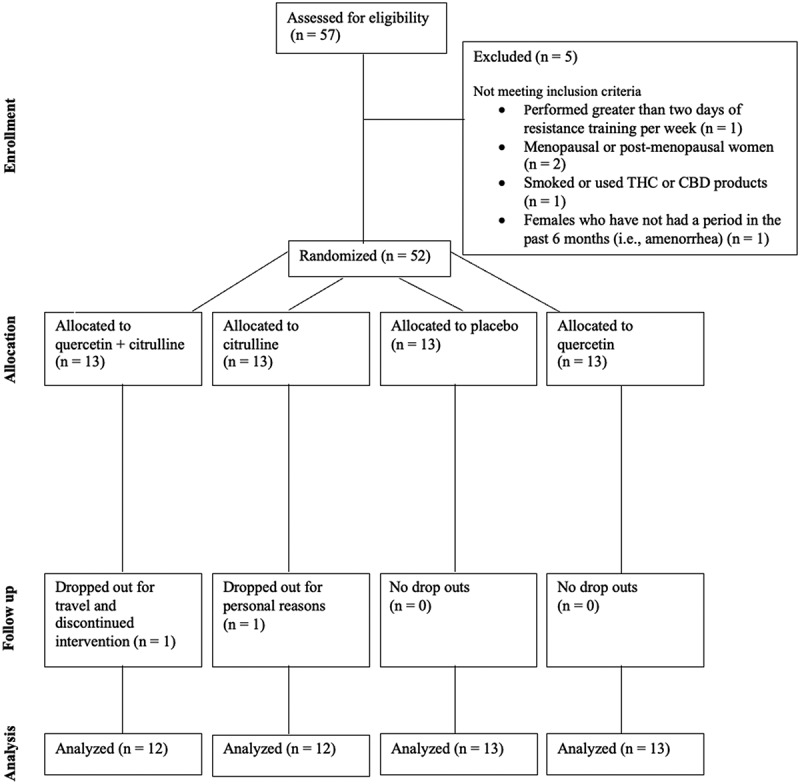
CONSORT participant flow diagram.

Participants included 50 males (*n* = 42) and females (*n* = 8) (ages 18–55 years) who regularly competed in category 1–3 cycling races across several disciplines, including mountain, gravel, cross country, road, and cyclocross. Figure 2 outlines subject recruitment, randomization, and reasons for dropout. Baseline anthropometric measures for participants randomized to QCT+CIT (*n* = 11 males, 1 female), QCT (*n* = 9 males, 4 females), CIT (*n* = 11 males, 1 female), and PL (*n* = 11 males, 2 females) groups are summarized in Table 1. No significant differences were found for age, gender, ethnicity, or anthropometric measures (*p* > 0.05). There were no significant changes in menstrual cycles among women. The dietary recall revealed no statistical significance within and between participants among groups. Total weekly training distance ranged from 101.58 ± 64.36 to 285.92 ± 92.10 km represented as mean ± SD, respectively. Cyclist’s total weekly time spent training ranged from 11.16 ± 5.08 to 18.55 ± 7.41 hours. There were no differences in PA across supplement groups (*p* > 0.05). Total cyclist performance measures are reported (Table 1, 2). Further, there was a 92% supplement compliance rate with all participants.

**Table 1.** Cyclist demographic characteristics at baseline familiarization testing, visit 1.

m ± SD	Total Suppl. Groups	QCT+CIT ± n = 12	QCT ± n = 13	CIT ± n = 12	PL ± n = 13
Age ± yr	30.37 ± 8.81	33. 43 ± 9.12	34.59 ± 8.94	36.54 ± 8.11	36.86 ± 9.56
Ht ± cm	175.75 ± 9.45	175.57 ± 12.70	172.96 ± 8.88	177.79 ± 8.19	176.84 ± 7.89
Lean Tissue ± cm	58.16 ± 7.90	59.42 ± 7.46	55.47 ± 10.15	58.97 ± 7.51	58.96 ± 6.21
Body Fat ± %	21.96 ± 6.18	20.87 ± 5.61	22.85 ± 5.93	23.28 ± 6.89	20.89 ± 6.58
Total Weekly Cycling Volume ± AU	1460.28 ± 580.48	1415.74 ± 368.13	1447.21 ± 419.10	1346.66 ± 813.88	1619.34 ± 587.02
N ± %					
Females	8 ± 16	1 ± 12.5	4 ± 50	1 ± 12.5	2 ± 25
Ethnicity N ± %					
White	40 ± 80	11 ± 27.5	10 ± 25	9 ± 22.5	10 ± 25
African American	3 ± 6	0 ± 0	0 ± 0	2 ± 67	1 ± 33
Asian	2 ± 4	1 ± 50	1 ± 50	0 ± 0	0 ± 0
Hispanic	5 ± 10	0 ± 0	2 ± 40	1 ± 20	2 ± 40

Data are presented as mean ± SD. Ht = height, VAT = visceral adipose tissue.

**Table 2.** Performance measures for baseline familiarization, pre-supplementation, and post-supplementation visits.

Variables	Supplement Groups	Baseline	Pre-Supplementation	Post-Supplementation	% ∆ change
Body Mass (kg)	QCT+CIT (*n*=12)	78.23 ± 12.29	78.14 ± 12.24	78.00 ± 12.06	−0.18%
	QCT (*n* = 13)	74.61 ± 13.70	74.81 ± 13.55	74.52 ± 13.16	−0.39%
	CIT (*n* = 12)	79.75 ± 8.29	79.86 ± 8.23	79.99 ± 8.72	0.16%
	PL (*n* = 13)	77.36 ± 7.44	77.62 ± 7.57	77.36 ± 10.41	−0.33%
	Total (*n* = 50)	77.43 ± 10.60	77.55 ± 10.55	77.40 ± 10.41	−0.19%
Average Power (Watts)	QCT+CIT (*n*=12)	245.94 ± 42.92	251.61 ± 48.35	255.17 ± 48.28	1.41%
	QCT (*n* = 13)	249.36 ± 49.08	253.23 ± 44.45	263.46 ± 52.73	4.04%
	CIT (*n* = 12)	238.13 ± 53.70	232.53 ± 52.55	239.00 ± 55.20	2.78%
	PL (*n* = 13)	232.88 ± 55.26	255.67 ± 52.97	248.08 ± 50.80	−2.97%
	Total (*n* = 50)	241.56 ± 49.42	248.51 ± 48.99	251.60 ± 51.02 †(*p* < 0.01)	1.24%
Max Power (Watts)	QCT+CIT (*n*=12)	462.17 ± 89.92	514.17 ± 129.97	529.00 ± 150.33	2.88%
	QCT (*n* = 13)	488.17 ± 164.78	484.54 ± 116.44	497.54 ± 111.03	2.68%
	CIT (*n* = 12)	560.17 ± 148.40	511.92 ± 128.99	533.67 ± 190.02	4.25%
	PL (*n* = 13)	459.54 ± 127.03	484.62 ± 99.07	485.54 ± 103.69	0.19%
	Total (*n* = 50)	491.82 ± 137.84	499.24 ± 116.44	510.64 ± 138.77	2.28%
Relative Average Power (Watts/kg)	QCT+CIT (*n*=12)	3.17 ± 0.65	3.27 ± 0.67	3.33 ± 0.74	1.83%
	QCT (*n* = 13)	3.38 ± 0.65	3.41 ± 0.62	3.59 ± 0.78	5.28%
	CIT (*n* = 12)	3.03 ± 0.72	2.89 ± 0.64	3.00 ± 0.66	3.81%
	PL (*n* = 13)	3.02 ± 0.83	3.34 ± 0.84	3.24 ± 0.74	−2.99%
	Total (*n* = 50)	3.15 ± 0.71	3.23 ± 0.71	3.29 ± 0.74	1.86%
Average Heart Rate (bpm)	QCT+CIT (*n*=12)	163.64 ± 15.66	164.49 ± 11.86	164.94 ± 10.17	0.27%
	QCT (*n* = 13)	168.38 ± 10.30	166.67 ± 10.86	168.08 ± 7.21	0.85%
	CIT (*n* = 12)	167.84 ± 10.62	170.08 ± 8.16	166.12 ± 12.28	−2.33%
	PL (*n* = 13)	157.39 ± 13.95	162.27 ± 12.31	164.09 ± 11.37	1.12%
	Total (*n* = 50)	164.37 ± 13.16	165.82 ± 11.00	165.82 ± 10.19	0.00%
Average VO_2_ (ml/kg/min)	QCT+CIT (*n*=12)	40.01 ± 6.72	42.61 ± 7.38	43.00 ± 6.02	0.92%
	QCT (*n* = 13) * (*p* = 0.03)	40.50 ± 7.18	41.92 ± 7.59	43.96 ± 6.89 †(*p* = 0.05)	4.87%
	CIT (*n* = 12)	38.56 ± 5.66	38.35 ± 5.87	40.31 ± 5.12 †(*p* = 0.04)	5.11%
	PL (*n* = 13)	40.56 ± 7.43	43.04 ± 8.00	41.08 ± 6.56	−4.55%
	Total (*n* = 50)	39.93 ± 6.64	41.52 ± 7.29	42.10 ± 6.19	1.40%
Highest Achieved VO_2_ (ml/kg/min)	QCT+CIT (*n*=12)	46.38 ± 6.86	48.50 ± 7.23	49.61 ± 5.83	2.29%
	QCT (*n* = 13)	45.69 ± 7.42	47.40 ± 7.68	47.96 ± 6.13	1.18%
	CIT (*n* = 12	43.40 ± 4.85	43.36 ± 5.24	45.04 ± 5.18	3.87%
	PL (*n* = 13)	45.78 ± 7.61	48.50 ± 8.51	45.78 ± 7.47	−5.61%
	Total (*n* = 50)	45.33 ± 6.69	46.98 ± 7.38	47.09 ± 6.31	0.23%
Average RER (au)	QCT+CIT (*n*=12)	1.00 ± 0.05	0.98 ± 0.43	0.97 ± 0.06	−1.02%
	QCT (*n* = 13)	0.97 ± 0.08	0.96 ± 0.06	0.96 ± 0.06	0.00%
	CIT (*n* = 12)	0.96 ± 0.07	0.97 ± 0.07	0.99 ± 0.05	2.06%
	PL (*n* = 13)	1.00 ± 0.08	0.97 ± 0.07	0.96 ± 0.05	−1.03%
	Total (*n* = 50)	0.98 ± 0.07	0.97 ± 0.07	0.97 ± 0.05	0.00%
Highest RER Achieved (au)	QCT+CIT (*n*=12)	1.08 ± 0.08	1.05 ± 0.07	1.04 ± 0.08	−0.95%
	QCT (*n* = 13)	1.04 ± 0.08	1.03 ± 0.08	1.02 ± 0.07	−0.97%
	CIT (*n* = 12)	1.03 ± 0.06	1.04 ± 0.09	1.07 ± 0.07	2.88%
	PL (*n* = 13)	1.08 ± 0.11	1.04 ± 0.06	1.02 ± 0.08	−1.92%
	Total (*n* = 50)	1.06 ± 0.08	1.04 ± 0.07	1.04 ± 0.07	0.00%
Average RPE (au)	QCT+CIT (*n*=12)	15.72 ± 1.53	15.80 ± 1.66	15.88 ± 1.33	0.51%
	QCT (*n* = 13)	15.82 ± 1.42	15.51 ± 1.26	15.65 ± 1.13	0.90%
	CIT (*n* = 12)	16.00 ± 1.57	15.97 ± 1.49	16.05 ± 1.28	0.50%
	PL (*n* = 13)	15.63 ± 1.37	16.01 ± 1.40	16.02 ± 1.52	0.06%
	Total (*n* = 50)	15.79 ± 1.43	15.82 ± 1.42	15.90 ± 1.29	0.51%
Highest RPE Achieved (au)	QCT+CIT (*n*=12)	18.58 ± 1.88	18.83 ± 1.85	19.58 ± 0.67	3.98%
	QCT (*n* = 13)	18.31 ± 1.49	18.77 ± 1.24	19.08 ± 0.95	1.65%
	CIT (*n* = 12)	19.08 ± 1.44	19.50 ± 0.90	19.17 ± 0.94	−1.69%
	PL (*n* = 13)	18.69 ± 1.18	19.23 ± 0.73	19.08 ± 1.04	−0.78%
	Total (*n* = 50)	18.66 ± 1.49	19.08 ± 1.24	19.22 ± 0.91	0.73%
Time Trial Performance (minutes)	QCT+CIT (*n*=12)	30.27 ± 2.35	30.48 ± 2.33	30.17 ± 2.23	−1.02%
	QCT (*n* = 13)	29.96 ± 2.36	30.15 ± 2.10	30.19 ± 2.59	0.13%
	CIT (*n* = 12)	30.93 ± 2.69	30.83 ± 2.26	31.19 ± 2.56	1.17%
	PL (*n* = 13)	30.82 ± 3.19	30.39 ± 2.34	30.74 ± 2.59	1.15%
	Total (*n* = 50)	30.49 ± 2.62	30.46 ± 2.20	30.57 ± 2.46	0.36%

Data are presented as mean ± SD. VO2 = oxygen consumption, RER = respiratory exchange ratio, RPE = rating of perceived exertion. † = within-group differences between visits. * = QCT > PL group. % ∆ change = change from pre-to-post supplementation.

### 3.1. Performance measures

#### 3.1.1. Time-trial (TT) completion time

**Figure 3.**
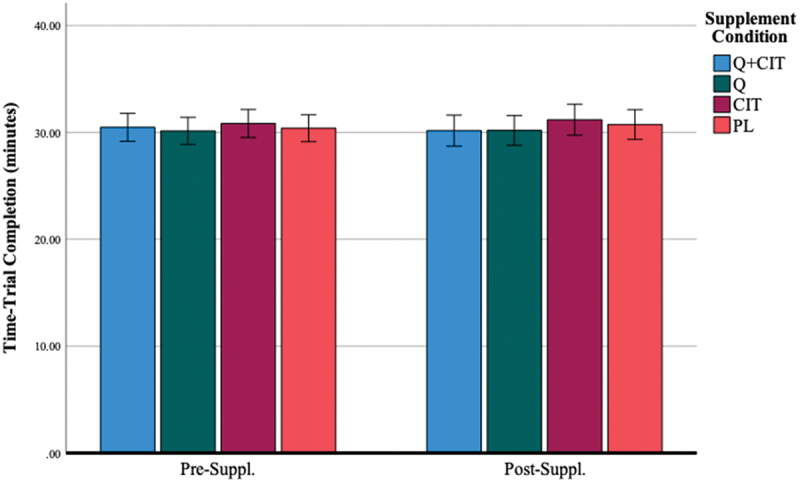
Time-trial completion before and after 28 days of supplementation.

Average TT performance was 30.50 ± 2.65 minutes, 30.46 ± 2.26, and 30.57 ± 2.49, at visits 1, 2, and 3, respectively. Total time trial performance did not differ among groups at all visits (Table 2). After 28 days of supplementation, TT performance did not change due to supplementation in any group (Figure 3). The main effect of the visit was non-significant [F (1, 46) = 0.43, *p* = 0.52]. The main effect of the supplement was non-significant [F (3, 46) = 0.31, *p* = 0.82]. There was a low variability between TT performance between visits: visit 1 CV = 8.6% [CI: 29.74–31.23], visit 2 CV = 7.2% [29.83–31.08], and visit 2 CV = 8.0% [CI: 29.87–31.27] and a strong test-rest validity between visit 1-visit 2 (ICC = 0.78).

*Data are displayed as means ± SEM.

#### 3.1.2. Average oxygen consumption (VO_2_)

**Figure 4.**
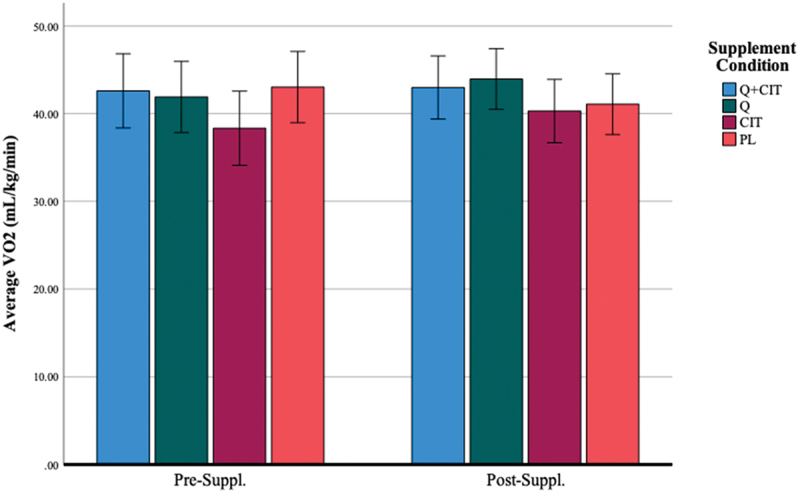
Average VO_2_ before and after 28 days of supplementation.

**Figure 5.**
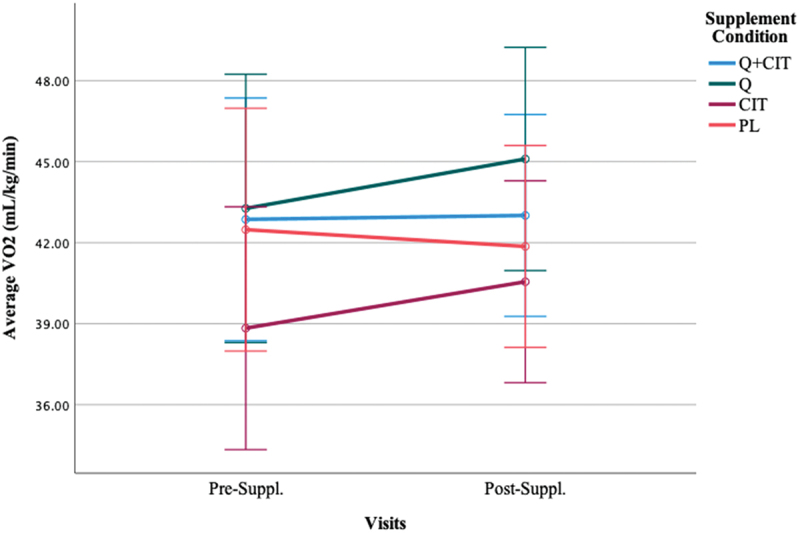
Average VO_2_ differences between supplement groups excluding women before (visit 2) and after 28 days of supplementation (visit 3).

Participants maintained an average VO_2_ of 39.96 ± 6.75, 41.48 ± 7.2, and 42.09 ± 6.15 (mL/kg/min) and their highest achieved oxygen consumption was 45.31 ± 6.69, 46.94 ± 7.17, and 47.10 ± 6.15 (mL/kg/min) at visits 1, 2, and 3 during the TTs, respectively (Table 2). The results did not achieve statistical significance in average VO_2_ nor highest achieved VO_2_ following supplementation (Figure 4) (*p* > 0.05). The main effect of the visit was non-significant [F (1, 46) = 1.32, *p* = 0.26]. The main effect of time was significant, indicating a large effect size [F (4, 46) = 143.61, *p* < 0.01, η^2^  = 0.76] such that VO_2_ at 0-km was significantly lower than all other distance markers (*p* < 0.00) and VO_2_ at 20-km was significantly higher than all other distance markers (*p* < 0.00). However, VO_2_ at 5–10- and 15-km did not significantly differ from each other at pre- (visit 2) and post-supplementation (visit 3) (*p* > 0.05). The main effect of the supplement was non-significant [F (3, 46) = 0.81, *p* = 0.49]. The interaction effect of visit*supplement was significant [F (3, 46) = 3.24, *p* = 0.03, η^2^ = 0.17]. The average VO_2_ differentially changed from pre-to-post-supplementation in QCT (*p* = 0.05) and CIT (*p* = 0.04) groups, but not in the QCT+CIT and PL groups. There was no difference in VO_2_ across collapsed groups from pre-supplementation (visit 2) to post-supplementation (visit 3) (Figure 5). However, significantly differed on average VO_2_ from each other (*p* = 0.03). When excluding women, the main effect of time was significant, revealing a large effect size [F (4, 38) = 121.28, *p* < 0.01, η^2^ = 0.76] such that VO_2_ at 0-km was significantly lower than all other distance markers (*p* < 0.00) and VO_2_ at 20-km was significantly higher than all other distance markers (*p* < 0.00).

*Data are displayed as means ± SEM.

*Data are displayed as means ± SEM.

#### 3.1.3. Average power output

**Figure 6.**
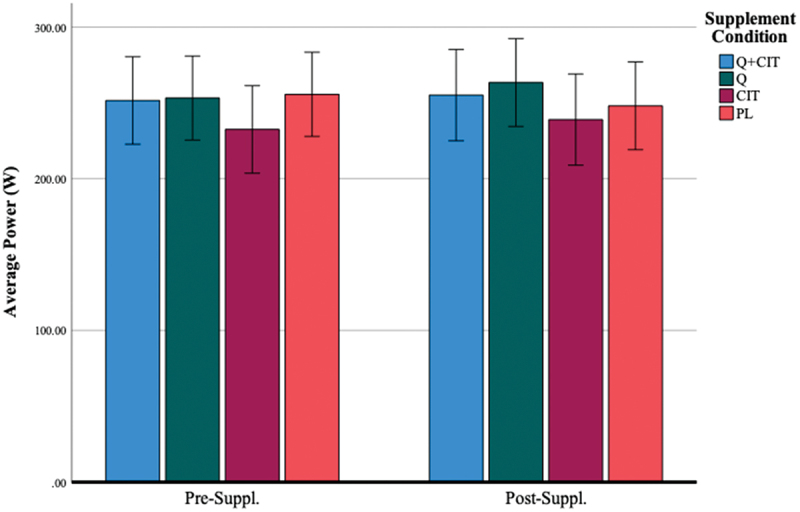
Average power before and after 28 days of supplementation.

**Figure 7.**
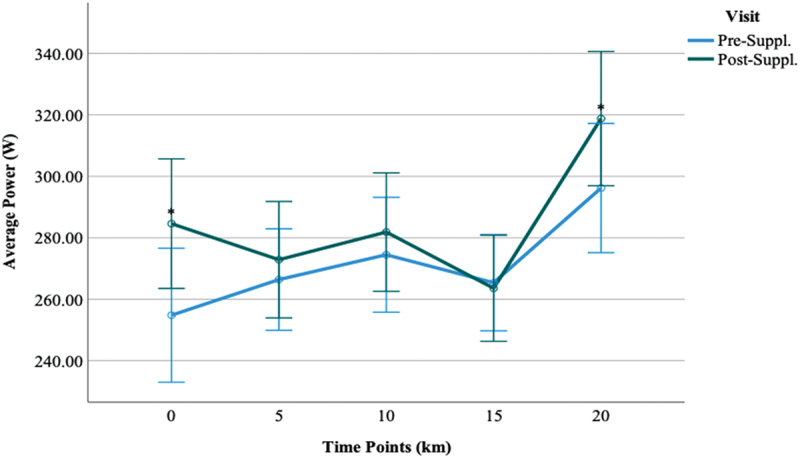
Comparisons of average power between visit 2 and visit 3 time-points.

Participants in all groups were able to maintain an average power output (Watts, W) of 251.61 ± 48.35 (QCT+CIT), 253.23 ± 44.45 (QCT), 232.53 ± 52.55 (CIT), 255.67 ± 52.97 (PL) at pre-supplementation (visit 2) and 255.17 ± 48.28 (QCT+CIT), 263.46 ± 52.73 (QCT), 239.00 ± 55.20 (CIT), and 248.08 ± 50.80 (PL) at post-supplementation 2), respectively. There were no statistical differences in average power output with supplementation pre-to-post-supplementation (Figure 6). The main effect of the visit was significant [F (1, 46) = 8.89, *p* = 0.01, η^2^ = 0.16]. The main effect of time was significant, signifying a large effect size [F (4, 46) = 15.69, *p* < 0.01, η^2^ = 0.25]. The main effect of the supplement was non-significant [F (3, 46) = 0.65, *p* = 0.59]. The interaction effect of visit*time was significant with a medium effect size [F (4, 46) = 3.12, *p* = 0.02, η^2^ = 0.06]. At 0 and 20-km time points, there was a difference in average power collapsed across groups between pre-to-post-supplementation (*p* = 0.01, 0.02, *d* = 0.77, 0.73) (Figure 7). When excluding women, the main effect of visit was significant with a large effect size [F (1, 38) = 9.28, *p* < 0.01, η^2^ = 0.20]. The main effect of time was significant with a large effect size [F (4, 38) = 11.30, *p* < 0.01, η^2^ = 0.23]. The interaction effect of visit*time was significant with a medium effect size [F (4, 38) = 2.87, *p* = 0.02, η^2^ = 0.07].

*Data are displayed as means ± SEM.

*Data are displayed as means ± SEM.

#### 3.1.4. Heart rate (HR)

**Figure 8.**
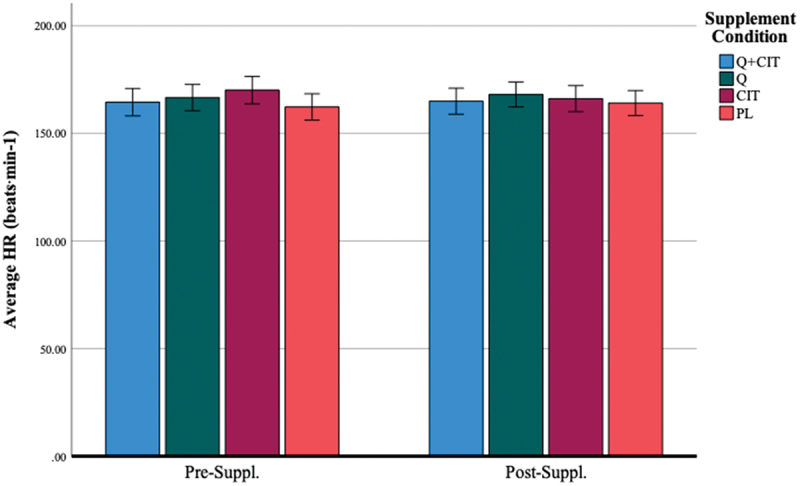
Average HR (beats⋅min-^1^) before and after 28 days of supplementation.

Average HR (beats⋅ min^−1^) during the trials were 164.49 ± 11.86 (QCT+CIT), 166.67 ± 10.86 (QCT), 170.08 ± 8.16 (CIT), 162.27 ± 12.31 (PL) at pre-supplementation (visit 2) and 164.94 ± 10.17 (QCT+CIT), 168.08 ± 7.21 (QCT), 166.12 ± 12.28 (CIT), and 164.09 ± 11.37 (PL) at post-supplementation (Table 2), respectively. The main effect of the visit was non-significant [F (1, 46) = 0.20, *p* = 0.61]. However, the main effect of time was significant, indicating a large effect size [F (4, 46) = 300.27, *p* < 0.01, η^2^ = 0.87]. HR significantly increased at all distance markers (0-, 5-, 10-, 15-, and 20-km) at pre- (visit 2) and post-supplementation (visit 3) (*p* < 0.01). The main effect of the supplement was non-significant [F (3, 46) = 0.78, *p* = 0.51] (Figure 8). When excluding women, the main effect of time was significant with a large effect size [F (4, 38) = 268.97, *p* < 0.01, η^2^ = 0.88] such that HR was significantly different at all distance markers at pre- (visit 2) and post-supplementation (visit 3) (*p* < 0.01).

*Data are displayed as means ± SEM.

#### 3.1.5. Respiratory exchange ratio (RER)

**Figure 9.**
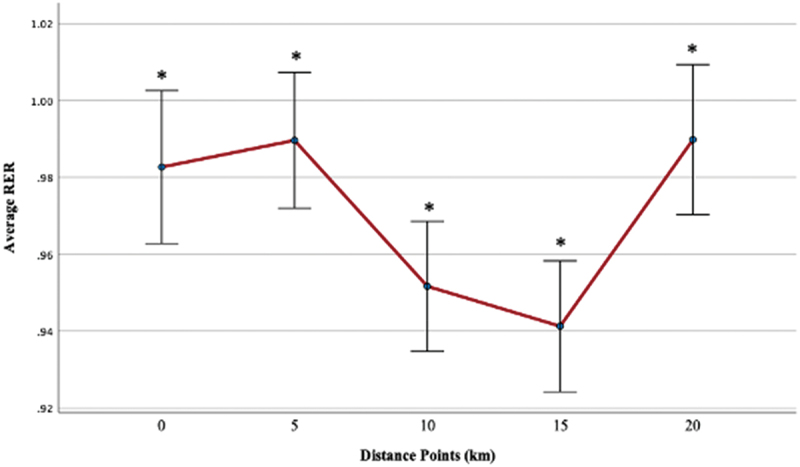
Average RER across distance markers (km) at pre-supplementation (visit 2) and post-supplementation (visit 3).

The average RER at each visit was 0.98 ± 0.07, 0.97 ± 0.07, and 0.97 ± 0.05, respectively. The respiratory exchange ratio (in the range between 1.10 and 1.15) indicated participants neared maximal effort [52–54]. The highest achieved RER at each visit was 1.06 ± 0.08, 1.04 ± 0.07, 1.04 ± 0.07, respectively. Regardless of the group, there were no statistically significant results from pre-supplementation to post-supplementation for the highest achieved RER (*p* > 0.05). The main effect of the visit was non-significant [F (1, 46) = 0.09, *p* = 0.77]. The main effect of time was significant, revealing a large effect size [F (4, 46) = 16.75, *p* < 0.00, η^2^ = 0.27] such that RER at 0 km was significantly lower than 10 and 15 km (*p* < 0.00), and RER at 20 km was significantly higher than 10 and 15 km (*p* < 0.00). RER at 5, 10, and 15 km significantly differed from each other at pre- (visit 2) and post-supplementation (visit 3) (Figure 9). The main effect of the supplement was non-significant [F (3, 46) = 0.32, *p* = 0.81].

When excluding women, the main effect of time was significant, demonstrating a large effect size [F (4, 38) = 13.56, *p* < 0.00, η^2^ = 0.26] such that RER at 0 km was significantly lower than 15 km (*p* < 0.00), and RER at 20km was significantly higher than 10 and 15 km (*p* < 0.00). RER at 5, 10, and 15 km significantly differed from each other at pre- (visit 2) and post-supplementation (visit 3).

#### 3.1.6. Ratings of perceived exertion (RPE)

The average RPE at each visit was 15.79 ± 1.43, 15.82 ± 1.42, and 15.90 ± 1.29. An RPE greater than 17 indicated participants neared maximal effort [52–54]. The highest achieved RPE at each visit was 18.66 ± 1.49, 19.08 ± 1.24, and 19.22 ± 0.91, respectively. Regardless of the group, the average RPE was non-significant (*p* > 0.05). The visit’s main effect was non-significant [F (1, 45) = 0.50, *p* = 0.49]. The main effect of time was significant, revealing a large effect size [F (4, 45) = 273.98, *p* < 0.00, η^2^ = 0.86] such that RPE significantly increased at all distance markers (0-, 5-, 10-, 15-, and 20-km) at pre- (visit 2) and post-supplementation (visit 3) (*p* < 0.01). The supplement’s main effect was non-significant [F (3, 45) = 0.26, *p* = 0.86].

**Figure 10.**
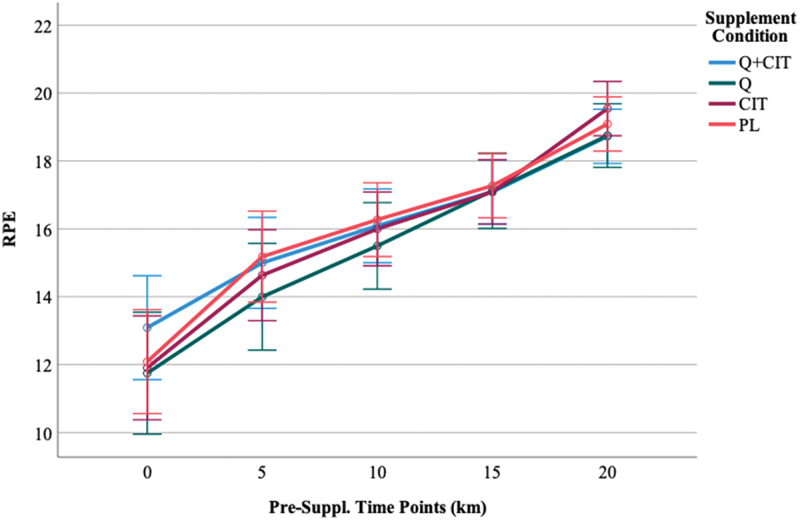
RPE time points at pre-supplementation, visit 2.

**Figure 11.**
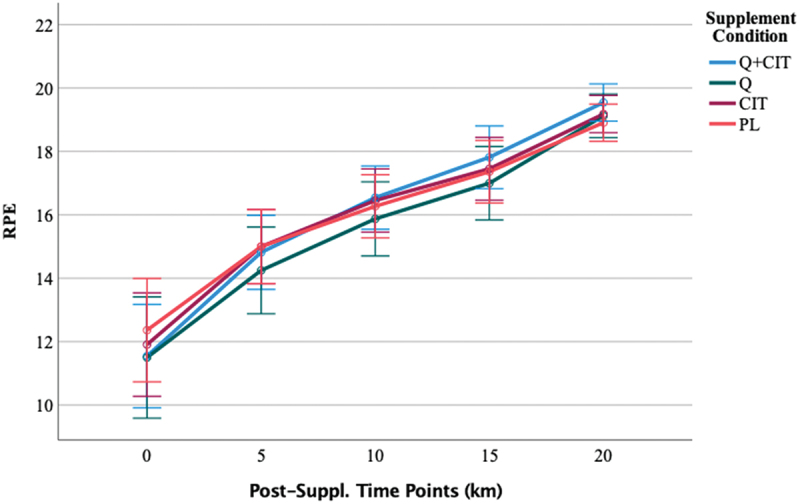
RPE time points at post-supplementation, visit 3.

When excluding women, the main effect of time was significant [F (4, 38) = 221.67, *p* < 0.00, η^2^ = 0.86] such that RPE was significantly different at all distance markers at pre- (visit 2) and post-supplementation (visit 3) (*p* < 0.01). The 3-way visit*time*supplement interaction was significant with a large effect size [F (12, 38) = 2.19, *p* = 0.02, η^2^ = 0.15]. Pairwise comparisons revealed the source of this 3-way interaction to be a significant difference in the QCT+CIT group at 0-km (*p* = 0.15) and at 20-km (*p*  = 0.01) from pre-to-post-supplementation. The interaction resulted in a higher RPE in the QCT+CIT at 0-km and lower RPE at 20-km pre-supplementation (Figure 10) and lower RPE at 0-km and higher RPE at 20-km at post-supplementation, compared to the other groups (Figure 11). No other group differences were detected from pre-to-post supplementation.

Data are displayed as means ± SEM.

Data are displayed as means ± SEM.

## 4. Discussion

The role of supplementation in cycling is of high interest to increase performance in competition and metabolic adaptations associated with training [1]. Our results demonstrated that QCT+CIT does not increase 20-km TT cycling performance. Nevertheless, the novelty of our experimental design provides a strong base for systematic replication (e.g. an increase in cycling duration or the addition of other polyphenols). Participants in the QCT group received 1000 mg/day for 4 weeks which did not result in an improvement in cycling TT performance. In contrast, a 6-week supplementation of QCT (600 mg) combined with vitamins and minerals improved 30-km TT performance in similarly trained male athletes [9]. However, their study contained QCT+caffeine which is a powerful ergogenic aid to improve aerobic performance [63], making it difficult to isolate QCT’s effects on TT performance. Our results are in concert with previous results obtained from 13 trained cyclists who were supplemented with 1000 mg/day of QCT +820 mg Vitamin C, 40 mg Vitamin B3, or the same vitamin supplement without QCT for 28 days. These cyclists did not improve their cycling time trial performance after performing a defined amount of work [64]. This suggests that QCT may increase cycling TT performance to a greater extent when combined with antioxidants, polyphenols, nutrients, flavonoids, fish oil, or isoquercetin than an amino acid (CIT) [10]. Participants in the CIT group received 3000 mg/day for 4 weeks which did not result in an improvement in cycling TT performance. In contrast to our findings, 2.4 g/day CIT improved 4-km TT performance in trained males by 1.5% after 7 days of consumption then 2.4 g 1 hr. Before the TT, implying that CIT supplementation may reduce the time to complete a TT with shorter distance durations [65].

No studies have been performed investigating QCT+CIT, QCT, and CIT on average oxygen consumption during a 20-km TT. Here, our findings suggest that QCT supplementation did not enhance TT performance, but the ergogenic aid did increase VO2 from pre-to-post supplementation. The performance results are in contrast to research investigating QCT supplementation (600 mg) with essential vitamins for 6 weeks in trained cyclists, which reported increased 30-km TT performance but saw no effects of QCT on the change in VO_2max_ percentage [9]. However, our results reveal that QCT is capable of enhancing muscle oxidative potential, but the mechanisms are not understood. Nevertheless, the impact of QCT on oxygen consumption in trained athletes still remains controversial [9,32,66,67]. A study of 40 trained cyclists provided 1000 mg/day of QCT for 3 weeks failed to show any group differences in oxygen consumption [68]. Similar to our study, the administration of a higher dosage, longer duration, or specific QCT supplementation timing may be required. The impact of QCT and CIT on oxygen consumption remains controversial, with our results showing an increase in VO_2_ from pre- to post-supplementation, particularly driven by female participants. Although we controlled for the follicular phase, this suggests potential menstrual cycle influences that warrant further investigation with larger sample sizes. Even though the follicular phase has low concentrations of estrogen and progesterone, our findings suggest there may be a menstrual cycle influence (i.e. follicular phase) on oxygen consumption with the ingestion of a supplement. Our results contrast previous research demonstrating an increase in oxygen consumption during the luteal phase [69–72]. However, these studies did not examine the role of QCT and CIT during the menstrual cycle phases. The phases of an ovulatory menstrual cycle and the extent of improvements in aerobic capacity likely vary among individuals, which could be significant on an individual level. Further, QCT and CIT may play a role in the different phases of the menstrual cycle to improve oxygen consumption. Future research needs to test these supplements with a larger sample size and to focus on the potential mechanisms of action on any observed change in functional aerobic capacity, and also on sport-specific indicators of performance in trained athletes. Thus, gender and hormone variations need to be considered when examining the influence of a supplement on oxygen consumption.

There was an improvement in average power over the 20-km TT marks from pre-to-post-supplementation in men across all groups (i.e. 0-, 5-, 20-, 15-, 20-km). Our data suggest hormonal considerations when testing power production. However, our sample size of women may not have been large enough to detect sex differences and power changes with the supplement. Therefore, future research needs to consider the different menstrual phases and their effect on power performance with a larger female sample size. Further, limited research exists examining the influence of QCT+CIT, QCT, and CIT on power performance.

The time*visit*supplement interaction for RPE when excluding women modifies perceived effort with QCT+CIT. Our results suggest that combining QCT and CIT may alter the perceived effort at the start (0-km) and the end of a race (20-km), indicating that the supplement may have increased the level of effort the participants could maintain during the TT. Previously, 3 weeks of QCT supplementation did not alter RPE in trained male and female ultramarathoners did not alter RPE before the Western States endurance run [73]. In contrast, 6 weeks of QCT supplementation combined with antioxidants, at any given RPE, power output was higher in trained male participants suggesting that QCT supplementation supports a perceptual effect in trained males [9]. Based on the present findings and conflicting previous results, we contend that it is premature to preclude any beneficial effects of QCT supplementation on perceptual responses during a self-paced, competitive TT event.

Several limitations should be noted. Athletes may still benefit from QCT+CIT or QCT, but it may take longer to see a performance increase, possibly due to higher baseline nutrient levels [74,75]. Since some athletes may have had higher starting levels of QCT or CIT, there could be an upper limit of absorption for QCT [59,76]. Future studies need to assess baseline QCT and CIT levels on various training stimuli to precisely analyze any impact on cycling performance [77]. Moreover, we did not standardize fluid intake, which could have affected the cardiovascular measurements. Future studies need to standardize and/or track fluid intake and hydration levels pre- and post-exercise [78,79]. Additionally, we restricted our participant pool to Tier 2 athletes. Unfortunately, defining a Tier 2 athlete is still ambiguous for trained cyclists [35]. Future research needs to continue to develop a classification framework for trained cyclists. As such, the present study results cannot be generalized to other people, such as sedentary, clinical individuals, or regularly active non-cyclists.

## 5. Conclusions

The novel aspect of this research is that it was the first study to examine the effects of four-week supplementation with QCT (1000 mg/day) + CIT (6000 mg/day), QCT (1000 mg/day), or CIT (6000 mg/day) on metabolic, cardiovascular, and performance changes in trained cyclists during a 20-km time trial (TT). The study found that while QCT + CIT did not significantly impact the TT performance, both QCT and CIT improved VO_2_. Discrepancies between our findings and existing literature could be due to factors such as population selection, dosing strategy, supplement combinations, and testing protocols. Additional research is needed to evaluate QCT + CIT, QCT, or CIT combined with other polyphenols or anti-inflammatory molecules to determine their effectiveness in improving endurance performance. It is also important to study the effects of these supplements on additional biochemical measures such as inflammatory, oxidative, and recovery markers post-exercise. Future research should focus on identifying flavonoid mixtures that provide optimal, measurable benefits for cycling performance and determining the best outcome measures, dosing regimen, and duration for QCT + CIT supplementation. The results of this study suggest that QCT + CIT does not enhance TT performance in trained cyclists.
